# Skewed Distribution of Circulating Activated Natural Killer T (NKT) Cells in Patients with Common Variable Immunodeficiency Disorders (CVID)

**DOI:** 10.1371/journal.pone.0012652

**Published:** 2010-09-09

**Authors:** Karina I. Carvalho, Karina M. Melo, Fernanda R. Bruno, Jennifer E. Snyder-Cappione, Douglas F. Nixon, Beatriz T. Costa-Carvalho, Esper G. Kallas

**Affiliations:** 1 Federal University of São Paulo, São Paulo, Brazil; 2 Division of Experimental Medicine, San Francisco General Hospital, University of California San Francisco, San Francisco, United States of America; 3 University of São Paulo, São Paulo, Brazil; New York University, United States of America

## Abstract

Common variable immunodeficiency disorder (CVID) is the commonest cause of primary antibody failure in adults and children, and characterized clinically by recurrent bacterial infections and autoimmune manifestations. Several innate immune defects have been described in CVID, but no study has yet investigated the frequency, phenotype or function of the key regulatory cell population, natural killer T (NKT) cells. We measured the frequencies and subsets of NKT cells in patients with CVID and compared these to healthy controls. Our results show a skewing of NKT cell subsets, with CD4+ NKT cells at higher frequencies, and CD8+ NKT cells at lower frequencies. However, these cells were highly activated and expression CD161. The NKT cells had a higher expression of CCR5 and concomitantly expression of CCR5+CD69+CXCR6 suggesting a compensation of the remaining population of NKT cells for rapid effector action.

## Introduction

Patients with common variable immunodeficiency disorder (CVID) share characteristics including hypogammaglobulinemia, impaired B cell function, cytopenia, a low frequency of naive CD4+ T cells, an increase in cellular activation, and a skewed distribution of circulating B cell subsets [1]–[6]. Other immunological defects in CVID patients include a reduction in the absolute numbers of natural killer cells [7], and defective functions of dendritic cells [8]. Around 30% of CVID patients develop autoimmune diseases [9], [10]. The complex immunological dysfunctions in this disease are still being elucidated.

NKT cells are lymphocytes that express a rearranged Vα14-Jα18 semi-invariant TCR, and recognize a glycolipid (for example the prototypic α-Galactosyl-Ceramide (α-GalCer), presented in the context of the non-classical MHC molecule, CD1d [11]. Upon receptor T cell (TCR) stimulation, NKT cells are able to rapidly secrete both Th1 and Th2 cytokines [12]. NKT cells are an integral component in the suppression of autoreactive T cells and the prevention of autoimmune diseases [13], due to their capacity to quickly release large amounts of interleukin 4 (IL-4), favoring Th2 responses [14]. By directing the T cell immune response towards either a Th1 or Th2 phenotype, NKT cells appear to regulate the development of certain autoimmune conditions [15]. Selective defects in NKT cell number and cytokine production are present in individuals affected by different diseases such as systemic lupus erythematosis, rheumatoid arthritis, human immunodeficiency virus-1 (HIV-1) infection, and pulmonary tuberculosis [16]–[20].

In mice, NKT cells can be detected most frequently in liver, bone marrow and thymus, and are less common in the spleen, lymph node, blood and lung. The recruitment of leukocytes into tissues is dependent on a series of adhesive and activation steps mediated by adhesion molecules and chemokine receptor interactions [21], [22]. These chemokine receptors are expressed by T cells with homing potential to nonlymphoid tissues and are highly associated with inflammation [23]. Most NKT cells express CCR2, CCR5, CCR6, CXCR3 and CXCR6 [23]. In humans, CXCR6 is expressed preferentially on CD4+ and CD8+ memory T cells [24]. While CXCR6 is expressed on more double negative compared to CD4+ CD1d-restricted T cells, it is possible that CXCR6 could play a role in NKT cell development or homing of these cells to the liver [25]–[27]. Activation of NKT cells with α-GalCer enhances T-dependent humoral immune responses against co-administered T-dependent Ag, and this involves interaction with CD1d-expressing B cells [28]. NKT cells can also help B lymphocyte responses, and mice immunized with proteins and α-GalCer develop antibody titers 1–2 logs higher than those induced by proteins alone [29].

Because of the important interactions of B cells with NKT cells, we measured the frequencies, chemokine receptor patterns, and *ex-vivo* effector functions of NKT cells in CVID patients compared with healthy controls. We hypothesized that NKT cells would be reduced in CVID patients, and that this would influence the pathogenesis of CVID. Our results show that NKT cells are circulating at the same frequency in the peripheral blood in CVID patients as healthy donors, but that there is a skewing of NKT cell subsets in CVID patients.

## Materials and Methods

### Subjects and sample collection

This study was reviewed and approved by the local Institutional Review Board (IRB, Comitê de Ética em Pesquisa da Universidade Federal de São Paulo). IRB-approved informed consent was signed from all participants. Diagnosis of CVID was established according to the criteria by the Pan-American Group for Immunodeficiency (PAGID). Eighteen healthy controls and seventeen CVID patients were selected at the Division of Pediatric Clinical Immunology located at the Federal University of São Paulo.

Peripheral blood mononuclear cells (PBMC) were isolated from volunteers by density-gradient sedimentation over Ficoll-Paque (Pharmacia Biotech, Uppsala, Sweden). PBMC were then washed two times in Hank's balanced salt solution (Gibco, Grand Island, NY). Cells were cryopreserved in RPMI 1640 (Gibco), supplemented with 10% heat-inactivated fetal bovine serum (FBS; Gibco), 50 U/mL penicillin (Gibco), 50 µg/mL streptomycin (Gibco), 10 mM L-glutamine (Gibco), and 10% dimethyl sulphoxide (DMSO, Sigma, St. Louis). Cryopreserved cells were stored in liquid nitrogen until used in the assays. At the time of the assay, PBMC were rapidly thawed in a 37°C water bath and washed in RPMI 1640 supplemented with fetal calf serum, 100 U/mL penicillin, 100 µg/mL streptomycin, and 20 mM L-glutamine (R10). Cells were counted, checked for viability, and re-suspended in R10 at 10^6^ cells/mL.

### Flow cytometry

The following monoclonal antibodies were used in the assays: CD3-peridin chlorophyll protein (PerCP) (clone SK7), CD8-allophycocyanin (APC) (clone SK1) and CD4-phycoerythrin–cyanine (PE-Cy7) (clone SK3), from BD Biosciences (San Jose, CA); CCR5-PE-Cy7 (clone 2D7/CCR5) and CD161-APC (clone DX12) from BD PharMingen (San Jose, CA); Vα24 phycoerythrin (PE) (clone C15), Vβ11-Fluorescein isothiocyanate (FITC) (clone C21) from Immunotech (BC); CXCR6-APC (clone 56811) and CD69-allophycocyanin cyanine-7 (APC-Cy7) (clone FN50). All the antibodies were used for cell-surface staining. NKT cells were defined using CD3 positive cells also double positive for Vα24 and Vβ11. NKT cells subsets were defined using two panels with combinations of the following antibodies: CCR5, CXCR6, and CD69 for chemokine and activation and CD4, CD8, and CD161 for T cells subsets. Fluorescence minus one (FMO) was used for gate strategy [30].

After thawing, cells were centrifuged at 300 x*g*, for 5 min and transferred into 96 V bottom well plates (Nunc, Denmark) in 170 µL of staining buffer (PBS supplemented with 0.1% sodium azide [Sigma] and 1% FBS, pH 7.4–7.6) with the surface monoclonal antibodies panel. Cells were incubated at 4°C in darkness for 30 minutes, washed twice, and re-suspended in 100 µL of fixation buffer (1% paraformaldehyde [Polysciences, Warrington, PA] in PBS, pH 7.4–7.6).

Samples were acquired on a FACSCanto, using FACSDiva software (BD Biosciences), and then analyzed with FlowJo software version 8.7 (Tree Star, San Carlo, CA). Fluorescence voltages were determined using matched unstained cells. Compensation was carried out with CompBeads (BD Biosciences) single-stained with CD3-PerCP, CD4-FITC, CD8-APC-Cy7, CD4-PE-Cy7, CD3-PE, and CD3-APC. Samples were acquired until at least 800,000 events lymphocyte gate.

### Statistical analysis

Groups were compared using non-parametric models; data are reported as median and interquartile range (IQR). Comparisons among groups were carried out using Mann-Whitney non-parametric test. *p* values were considered significant if <0.05.

## Results

### Demographic data

The demographic, clinical and laboratory characteristics of participants are detailed in Table 1. The median age of the healthy controls was 23 years (IQR, 21–29), and for CVID subjects 26 years (IQR, 19–35). Sixty-one percent of healthy controls and 59% of CVID patients were female. No patient presented with an acute infection at the time of the study.

**Table 1:** Demographic, clinical and laboratory characteristics of control and CVID patient groups.

	*Controls*	*CVI patients*
	(n = 17)	(n = 17)
**Demographics**		
Age (median, IQR 25^th^,75^th^, in years)	23 (21−29)	26 (19−35)
Gender (female %)	61%	59%
Age at the diagnosis (median, IQR, in years)	–	22 (13.26)
Age at first symptoms (median, IQR, in years)	–	12 (3.16)
Average between initial symptoms and the diagnosis (in years)	–	8
**Clinical findings**		
Recurrent infections		
Pneumonia	–	15 (88.0%)
Otitis	–	6 (35.2%)
Sinusitis	–	11 (64.7%)
Chronic diarrhea	–	5 (29.0%)
Auto-immune diseases		
Hemolytic anemia	–	3 (17.64%)
Hypothyroidism	–	2 (11.7%
Hepatitis	–	1 (5.9%)
Chronic pulmonary diseases		
Bronchiectasis	–	7 (41.7%)
Atelectasis	–	3 (17.6%)
Bronchiolitis	–	2 (11.7%)
**Laboratory findings**		
Leucocytes (median, IQR, in cells/µl)	6770 (5710−7585)	7120 (5995−9655)
Neutrophyles (median, IQR, in cells/µl)	3205 (2820−3763)	4242 (3697−6370)
Lymphocytes (median, IQR, in cells/µµl)	2149 (1872−2796)	1715 (1196−2258)
Monocytes (median, IQR, in cells/µl)	411 (350−556)	533 (296−671)
CD3+ cells (median, IQR, in cells/µl)	1690 (1187−1861)	1301 (1018−2127)
CD4+ T cells (median, IQR, in cells/µl)	884 (675−1017)	604 (478−1064)
CD8+ T cells (median, IQR, in cells/µl)	556 (366−619)	606 (471−846)
Vα24+Vβ11+ NKT cells (%)	0.16 (0.054−0.275)	0.11 (0.045−0.320)
Vα24+Vβ11+ NKT cells (median, IQR, in cells/µl)	0.006 (0.001−0.011)	0.000 (0.00−0.001)
**Serum Ig levels (median; mg/dl)**		
Before treatment		
IgG	–	140 (24.10−630)
IgA	–	6.67 (5−24.3)
IgM	–	13.5 (8−17)
After treatment		
IgG	–	615 (482−1047)
IgA	–	5 (5−20)
IgM	–	8 (5−20)

IQR: interquartile range.

### Measurement of NKT cell frequencies in peripheral blood

**Figure 1:**
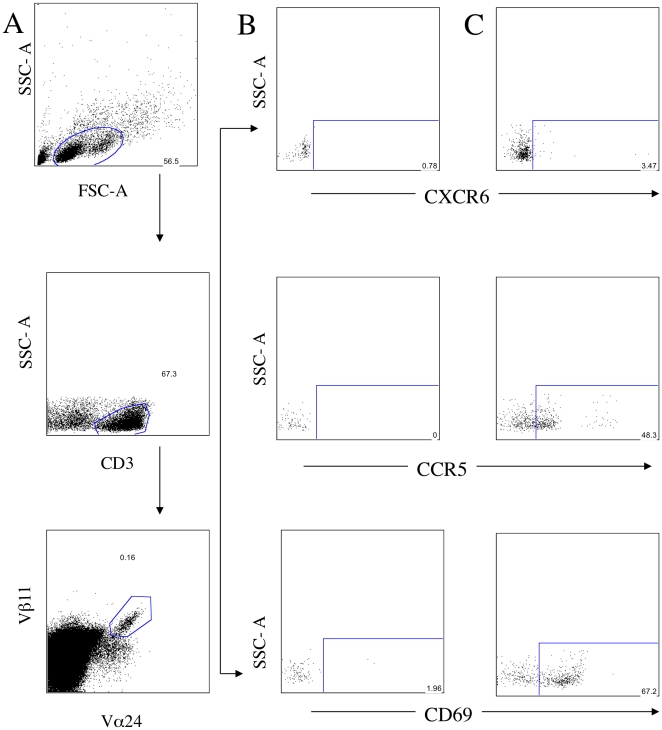
Expression of NKT cells in peripheral blood. (A) Representative flow cytometric analyses on PBMC, lymphocytes, CD3+ T cells and Vα24+Vβ11+ for NKT cells. (B) Fluorescence minus one (FMO) was used for gate strategy for CXCR6, CCR5 and CD69 in NKT cells. (C) Representative flow cytometric analyses on NKT cells in CVID patients. Comparisons among groups were carried out using the Mann-Whitney non-parametric test.

To identify NKT cells in circulation, we stained PBMC with monoclonal antibodies against anti-CD3, anti-Vβ11, and anti-Vα24, CD161, CD4, CD8, CCR5, CD69, and CXCR6, and analyzed the cells by six-color flow cytometry. NKT cells were identified by CD3+ and co-expression of Vβ11 and Vα24 (Figure 1A)[18]. We measured the frequency of NKT cells in both healthy controls and CVID patients. Due to the variability of NKT cell frequencies and limitations of available PBMC, data were included in this study if greater than 30 events were collected within the NKT gate. There was no significant difference in the frequency; on the other hand, the absolute number is increased in the healthy group [0.006 (0.001–0.011)] compared to CVID patients [0.000 (0.00–0001)], p = 0.0003 of circulating NKT cells in peripheral blood (Table 1).

### Circulating NKT cells of individuals with CVID have distinct chemokine receptor profiles compared with healthy controls

**Figure 2:**
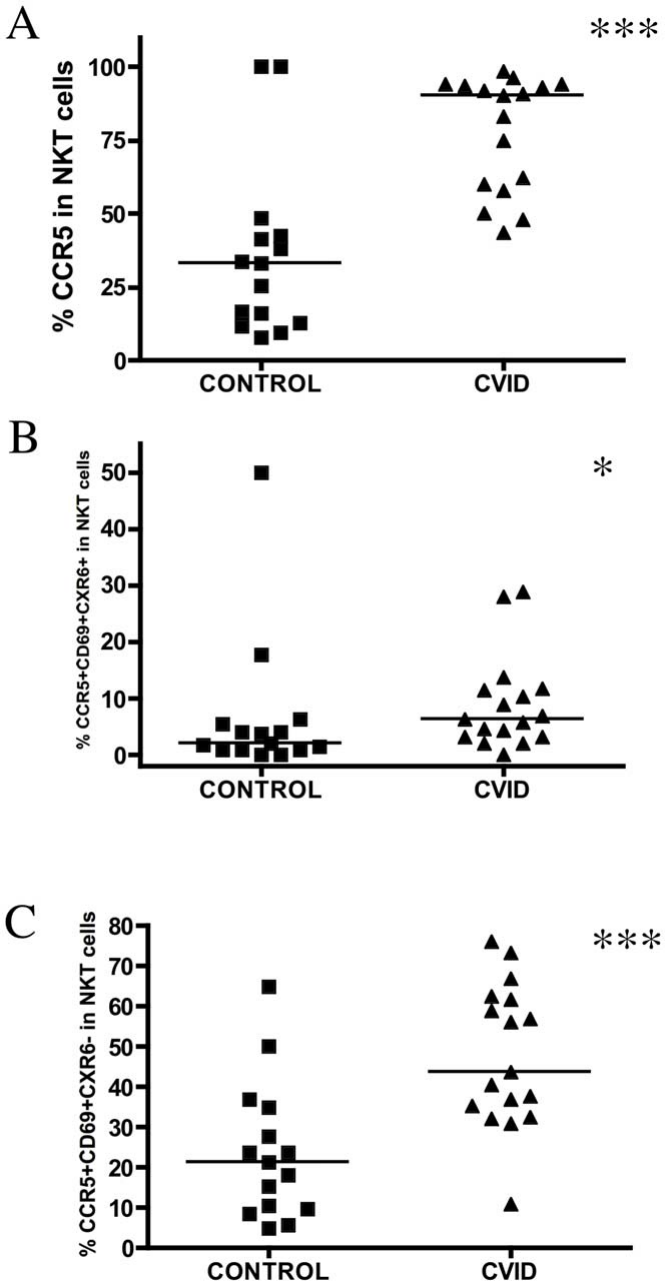
Percentage of activation, chemokine receptors in NKT cells. (A) Percentage of chemokine receptor CCR5 in NKT cells gate (Vα24+Vβ11) in representative healthy subject and CVID patient (p<0.0001). (B) Percentage of chemokine receptors CXCR6, CCR5 and CD69 marker in NKT cells (p<0.001). (C) Percentage of chemokine receptor CCR5 and CD69 marker in NKT cells (p<0.001). Comparisons among groups were carried out using the Mann-Whitney non-parametric test.

Next, we measured the surface expression of CXCR6 and CCR5 chemokine receptors on NKT cells in order to identify homing markers and activation status, respectively (Figure 1B,C). We observed that a higher percentage of NKT cells expressed CCR5 in CVID patients [90.10 (58.80–93.90)] when compared with healthy controls [33.30 (12.80–42.30)], p = 0.0006 (Figure 2A). NKT cells in CVID subjects had a higher concomitant expression of CCR5+CD69+CXCR6+ compared with healthy controls [1.910 (0.9000–5.440)], p = 0.03 (Figure 2B). The CCR5+CD69+CXCR6- fraction was also markedly altered in CVID [43.50 (33.70–62.00)] when compared with healthy controls [21.10 (9.680–34.90)], p = 0.0008 (Figure 2C). We observed a tendency for CVID patients to have a higher expression of CD69 on NKT cells compared to healthy subjects, but this did not reach a level of statistical significance.

### Expression of NKT cells subsets

**Figure 3:**
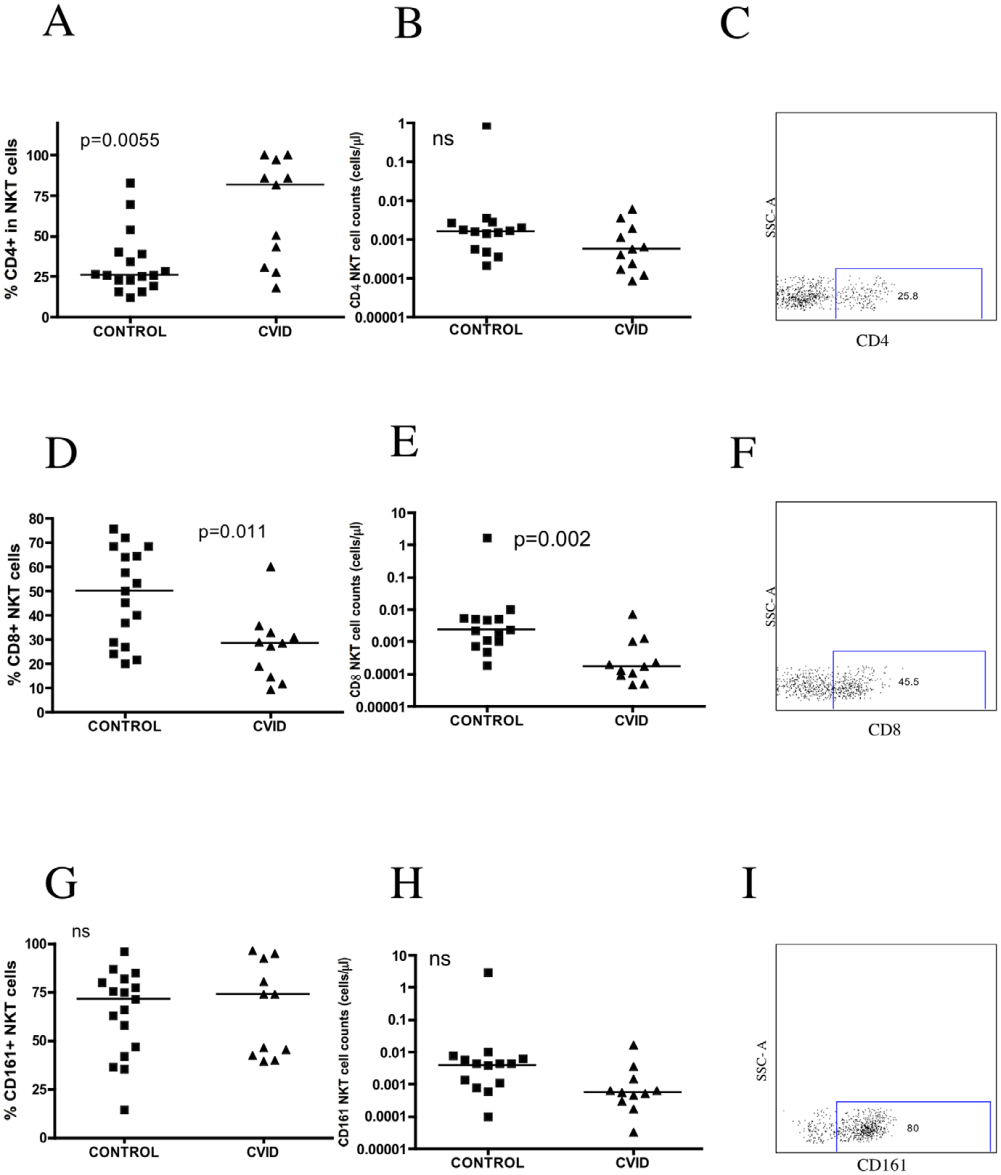
Subsets of NKT cells from CVID patients. (A) Percentage of CD4 marker in NKT cells (left) (p = 0.0055). (B) Absolute number of CD4 marker in NKT cells (middle). (C) Representative flow cytometry dot plot of CD4 marker (right). (D) Percentage of CD8 marker in NKT cells (left) (p = 0.011). (E) Absolute number of CD8 marker in NKT cells (middle) (p = 0.002). (F) Representative flow cytometry dot plot of CD8 marker (right). (G) Percentage of CD161 marker in NKT cells (left). (H) Absolute number of CD161 marker in NKT cells (middle). (I) Representative flow cytometry dot plot of CD161 marker (right).

CVID subjects had normal absolute numbers of CD4+ and CD8+ T cells (Table 1). We observed a higher expression of CD4+ NKT cells in CVID patients when compared to healthy controls [81.40 (30.80–97.00), and 26.10 (20.95–39.55), respectively, p = 0.0055](Figure 3 A,B,C), although these appeared to cluster in a high CD4+ expression group, and a lower CD4+ expression group. However, the absolute number of CD4+ NKT cells was not significantly different comparing to controls (Figure 3E). We also observed a lower expression of CD8+ NKT cells in CVID subjects when compared to healthy controls [28.60 (14.30–32.70), and 50.10 (27.70–66.45), respectively, p = 0.011] (Figure 3 D,E,F). These results were confirmed when we calculated for the absolute number of CD8+ NKT cells in CVID subjects and compared to healthy controls [0.000 (0.000–0.001) and 0.002 (0.000–0.005), respectively, p = 0.002] (Figure 3H).

### Expression of CD161 on NKT cells

**Figure 4:**
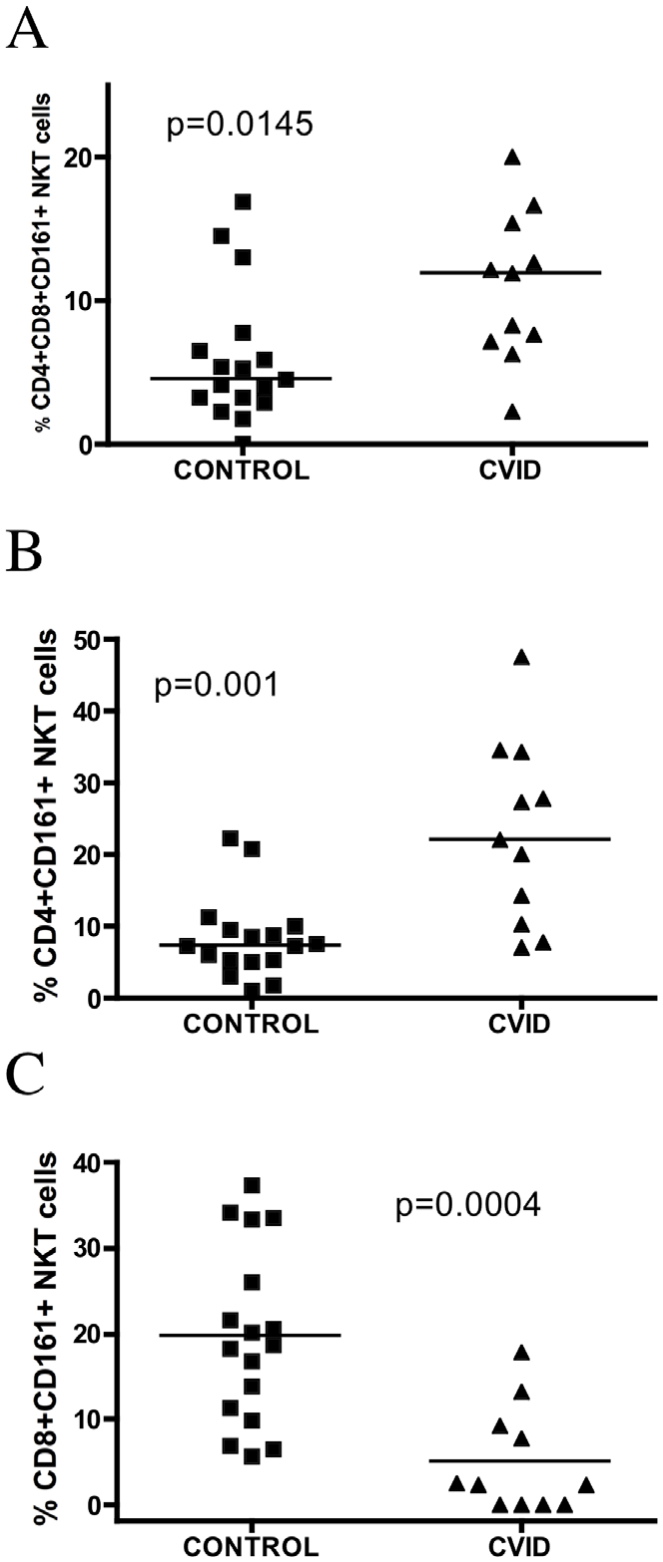
Subsets of NKT cells. (A) Percentage of CD161, CD8 and CD4 markers in NKT cells (p = 0.0145), (B) Percentage of CD4 and CD161 marker in NKT cells (p = 0.001). (C) Percentage of CD8 and CD161 markers in NKT cells (p = 0.0004).

CD161 is a marker commonly found on NK cells, and a maturation marker for NKT cells [31]. The percentage of CD161 expression and absolute number on NKT cells was not significantly different between healthy and CVID subjects (Figure 3 G,H,I). NKT cells had a higher expression of CD4+CD8+CD161+ in CVID patients when compared with healthy controls [11.90 (7.140–15.40), and 4.580 (3.035–7.170), respectively, p = 0.0145] (Figure 4A). However, the CD4+ and CD8+ NKT cells were heterogeneous in their expression of CD161. CVID patients expressed higher levels of CD4+CD161+ on NKT cells when compared to healthy donors [22.00 (10.40–34.20), and 7.340 (5.185–9.765), respectively, p = 0.0014] (Figure 4B). In contrast, CVID subjects had lower levels of CD8+CD161+ NKT cells compared to healthy donors [2.380 (0.000–9.200), and 18.70 (10.60–29.65), respectively, p = 0.0004] (Figure 4C).

## Discussion

In this study we examined the phenotype, activation, homing and maturation markers of NKT cells in patients with CVID. To our knowledge, this is the first study of the NKT cell subsets in patients with CVID. Recent data indicates that NKT cells were decreased in CVID patients [32]. Our results support previous observations of a decrease in the absolute number of NKT cells in CVID patients compared to healthy controls. It has been speculated that the low NKT numbers in CVID patients may play a role in the deficient humoral responses [32]. However, it could also be associated to impaired innate immune response, with implications in the susceptibility to opportunistic diseases. Indeed, the NKT cell subsets are skewed, and CD4+ NKT cells circulate at a higher frequency, and CD8+ at lower levels. All subsets of NKT cells were vastly activated and expressed high levels of CD161. Vα24^+^Vβ11^+^ NKT cells had a higher expression of CCR5, mostly with the CCR5+CD69+CXCR6- phenotype.

NKT cells appear to be important in the regulation and development of certain autoimmune conditions, and this could be related to defective signals that up-regulate CD1d expression [13], [15]. CD1d molecules are well conserved in evolution, and the limited degree of polymorphism in their genes makes them interesting targets for modulation of immunity in the prevention and treatment of human disease [33]. NKT cells can have multiple effects on an immune response, including the activation, regulation and attraction of innate immune cells, tolerance. NKT cells are selectively lost from circulation in HIV-1 infection, rheumatoid arthritis, and acute virus infections [16]–[19].

Sandberg et al. described that circulating NKT cells in healthy subjects were diverse in their expression of CD4 and CD8. Their results indicated that CD4+ NKT cells preferentially circulate through the lymph nodes and CD4- NKT cells go to peripheral tissues[31]. These results are in contrast to CVID patients, in whom CD4+ NKT cells were more frequent in the peripheral blood as opposed to CD8+ NKT cells. This higher expression of CD4+ NKT cells could potentially protect these patients from opportunistic mycobacterial infections and could impact autoimmunity.

NKT cells can express CD161 (NK1.1 in mice), activation marker for NK cell. Berzins et al. describe that NKT cells from thymus were CD161-, in contrast with adult peripheral blood, suggesting that CD161 expression is also a maturation marker for NKT cells in humans[34]. Consistent with that study, we found that CD4^+^ and CD8^+^ NKT cells were matured in CVID patients.

Developing Th1 cells acquire the capacity to produce IFNγ and expression of chemokine receptors such as CCR5, CXCR3, and CXCR6 that drive them to sites of delayed-type hypersensitivity reactions [35]. CXCR6 expression is associated with the function and fate of NKT cells by controlling their survival, cytokine production, and ability to induce tissue damage [36]. Previous studies describe that murine NKT cells were able to express CXCR6 [26], [36]. Interestingly, this chemokine receptor is expressed in humans on Th1 and Tc1 memory CD4+ and CD8+ T lymphocytes [24], and CXCR6 are preferentially expressed on double negative and CD8+ subsets of NKT cells [22], [23]. CXCR6 is expressed at a high level on NKT cells even under physiological conditions, as compared to other lymphocytes [37]. Our results revealed that the NKT cells were able to express higher levels of CCR5, mostly with the CCR5+CD69+CXCR6- phenotype. More studies need to address the function of these cells in CVID, and these studies could serve as a model to better understand the role of NKT cells in the immune response.

There are some limitations to this study. It is cross sectional, and NKT cell frequencies may change over time, although we have previously shown a stability of NKT cell numbers in healthy individuals [38]. CVID represents a spectrum of diseases, and different genetic causes might lead to differences in NKT cell expression. We sampled NKT cells only in peripheral blood. Despite these limitations, this is the first set of results to assess NKT cell frequency in CVID patients. Further studies are needed to clarify whether the increase of maturation, homing, and activation in NKT cells in CVID patients could be a counterbalance for the impaired the B cell function.

In summary, CVID subjects have a skewed fraction of activated homing NKT cells in peripheral blood. Boosting of NKT cell numbers through therapeutic modulation might be a valuable adjunctive treatment in CVID subjects.
